# Medicine and surgery for intermediate uveitis

**DOI:** 10.5935/0004-2749.202100113

**Published:** 2021

**Authors:** Jagdeep Singh Gandhi

**Affiliations:** 1 Worcester Royal Eye Unit, Worcestershire Royal Hospital, Worcester WR5 1DD, United Kingdom

Dear Editor,

Medicine and surgery are invokable when treating intermediate uveitis (IU). The two strategies have been appraised latterly^(1)^. The ensuing tract merges into the debate on therapeutics.

Down the slit-lamp the diagnosis of IU is usually straightforward. Such ocular inflammation can be induced by certain microbes. Examples are syphilis and tuberculosis. IU can also be the ocular component of certain diseases and disorders. For example, sarcoidosis can inflame any organ, and the intermediate uvea is merely one site. In some areas of the world IU is seen in patients with multiple sclerosis. Thus, as with other uveitides, IU may be confined to the eye or occur in parallel with systemic pathology.

In spite of the varied links there are times when no cause for the IU is detectable. One then assumes that an autoimmune axis is driving the clinical picture of uveitis. Not to be forgotten is that, in the patient aged 65+, the onset of IU is atypical. It is possible to misdiagnose intraocular lymphoma as a case of “IU”^(2)^.

Non-infective IU is the focus of the present discussion. Vitritis and macular oedema are its main sequelae. The mildest grades of unilateral or bilateral IU may not require any treatment. Here the vitreous is essentially clear and the eye is free of uveitic macular oedema. Tied to such anatomical bliss is superb visual function.

In devising therapy, it is seen whether the IU affects one or both eyes. For one-eyed IU the norm is local injection of steroid. Mild IU is treatable with sporadic doses of periocular steroid. But one-eyed IU that burns bright needs stronger therapy. Intravitreal steroid is used, but its cost can be forbidding. All intravitreal steroid implants are expensive. Long-acting steroid implants carry a huge price^(3)^. Preservative-free Triamcinolone is a low-cost intravitreal, but it has a short therapeutic action.

When starting drugs the disease activity is not predictable over a long clinical course. So a doctor commences treatment according to the severity of the baseline disease. For example, a steroid implant is injected at baseline even though later in the course the uveitis may have spontaneous phases of remission where no anti-uveitis therapy is actually required.

For one-eyed IU of high activity, it may not be possible to afford a long-acting steroid intravitreal. But it is onerous to inject short-acting intravitreal steroid every 3 to 6 months. In a young patient there is space for adding a systemic immunosuppressive. After all, the goal is to safeguard the major organ that is the eye. Compare with how a kidney transplant is protected with immunosuppression. I have seen Methotrexate used in a child with chronic anterior uveitis in one eye. The effect was a dramatic reduction of topical steroid usage and the related side-effects. The tradition is to employ systemic immunosuppression for bilateral uveitis, but it is seen how such therapy is sometimes usable for unilateral IU, so as to reduce the burden of local steroid injections.

At times the desirability of local steroid is clearcut. Take reproduction in young patients. Systemic immunosuppressives are intuitively undesirable around and during pregnancy. Hence one would prefer to dose the eye directly. Obesity is another scenario of challenge. Not enough drug-action may reach the eye via systemic dosing. To bypass the systemic resistance the IU is treated with local therapy.

On facing chronic IU in both eyes the mind turns to systemic immunosuppression. It is observable that immunosuppression can “cure” uveitis^(4)^. Not in every case, obviously, since uveitogenic immune activity is not always eradicable via the immunosuppressives that are in common use^(5)^. More potent immunosuppressives will extinguish the uveitis, but are potentially cancer-causing drugs^(6)^.

Experience shows that cases of bilateral IU will enter drug-free quiescence within 5 years. After starting with steroid, the steroid-sparer, once added, is the heart of the regimen. As the steroid is tapered off, slowly, the IU may be controllable with steroid-sparer alone. The next plan is to taper the steroid-sparer in small steps. For example, 2 years of full-dose and 3 years of reducing-dose Mycophenolate can coax IU into a stable and drug-free state.

Stronger therapy is needed for a severe form of bilateral IU. Its vitritis can simmer away on the popular recipe of minimal oral steroid and a steroid-sparing agent. But the prospect of heavier systemic suppression is unattractive. To control the fraction of uveitis activity that persists despite systemic therapy the clinician opts for local steroid. An allied aspect is that of asymmetrical disease. Only one eye may require the local adjunct.

Combined local and systemic therapy has two effects. The systemic arm blunts the underlying immune mechanism. Over time the uveitis is thus changed into a milder disease. To preach again the sermon of C. Stephen Foster: use non-steroid immunosuppression to “reset” the immune system and strive to obtain a drug-free cure for the patient^(4)^.

The local arm, as doses of local steroid, finely controls the uveitis. In clearing all vitritis and macular oedema the local therapy maximises vision. Smaller amounts of systemic drug and local drug are needed when systemic and local routes are yoked together to control the disease. Thus the side-effects of the respective routes are reduced.

Meanwhile, to avoid the side-effects of oral steroid, a doctor can now elect to combine a steroid-sparing immunosuppressive with an intravitreal implant. As already stated the aim is: tight control of the uveitis via the local agent, and an earlier drug-free remission via the immune-system-altering effect of the systemic agent.

Turning to surgery, the operation of posterior vitrectomy has the role of rescue. Some extreme cases are memorable. I had a 24-year-old patient with one-eyed IU ascribed to HLA-B27^(7)^. Vitritis totally obscured the fundus and was resistant to intensive steroid. The sight returned after a vitrectomy by a retinal surgeon. Find also a 63-year-old patient with multiple sclerosis and attendant chronic IU. Recurrent uveitic macular oedema is the pattern. But the wish is to avoid steroid injections, as well as systemic suppressives. Vitrectomy would be the way to quieten the uveitis within these constraints.

Systemic immune dynamics, however, continue after vitrectomy. Clinically, the eye may again show vitritis and macular oedema. Post-vitrectomy uveitis is more likely in the young with their fiercer inflammation. But excision of the vitreous will certainly subdue the uveitis^(8)^. In fact, for older patients a vitrectomy can cure the IU. That is, post-operative immune activity in older patients may have little or no clinical significance in the eye.

In conclusion, the risk-benefit ratio is gauged before pursuing anti-uveitis drugs or vitrectomy. Decisions are guided by factors such as age, one or both eyes, severity of disease, anticipated course, tolerance of drugs, comorbid conditions, and economics. By pivoting on scientific logic, pragmatism, and tailored care, a clinician can work through the challenges.
